# Nurse Marion's Romance

**Published:** 1887-05-07

**Authors:** Kate Furley


### Nurse Marion's Romance.

By Kate Furley.

Chapter VII.—The Acme of Selfishness.

Marion had to pause for a long time before she could muster sufficient courage to enter Holtayne's ward. A new revelation of his character had come to her with Mrs. Durrant's visit, but it was not this further *désillusionement* that hurt her most. It stung her, indeed, but only in connection with herself; she would have cared little for the selfishness Jack had displayed if she had been free from self-reproach. He had wounded her pride, which under the blow rose up in scorn of the vanity and weakness which had so befooled her. She was sorely tempted not to visit him again, and to ask to be relieved of her charge; and it was inevitable that any further intercourse between them should be of a disagreeable nature. But pride came to the rescue in this, and made her feel that it would be cowardice in her to avoid Holtayne now.

"And it would make him think that I *cared*, which is just what would please his vanity best," she told herself, as she went to see him.

Jack had been allowed to get up that day in honour of his visitor, and was now sitting by the fire. His well-shaped head with its clear-cut features rose above a crimson dressing-gown, on which the firelight brought out a hundred varying ruby and flame-coloured tints. The red light fell on his face, and covered its pallor with a manlier bronze, and seemed to find a kindred light in his dark eyes as he turned them towards the nurse when she entered.

He looked like some old picture by Velasquez or Vandyke. For a moment Marion paused, touched still by his beauty; but immediately an indignant revulsion came over her that she should still be moved by mere physical charm. Then she thought of how Holtayne's good looks were employed, and anger deepened into scorn. "'My face is my fortune' is a motto pardonable only in a woman," she said to herself.

"I am glad you have come, Marion," he said, in a voice that betrayed some weariness. "I feared I was not to get another glimpse of you. That woman who was here to-day tired me dreadfully, and I want your society to rest me—you are always restful."

"Mrs. Durrant seems to be anxious about you," answered Marion, coldly. "It is not every friend who would follow you from India so promptly, and be ready to undertake the care of a rather exacting invalid. I think you should speak more gratefully of her."

Jack cast an inquiring glance at her, as if to discover how much she knew; but merely said, in a matter-of-fact tone, "So you had a talk with her. What did she say?"

"She asked about your health, and when she was satisfied on that point she told me a little about herself."

"About herself and me?"

"No."

"But you seem to have inferred something from her manner; I can see it. What are you thinking?"

"Mrs. Durrant's manner is not favourable to reticence, but I see no reason for my having any thoughts," said Marion, keeping her eyes fixed steadily upon him.

He turned his away from her.

"I see you understand how things are," he replied, "at least, as far as externals go. Sit down by me, and let me explain it rightly to you."

She did as he asked, feeling, to her own surprise, no emotion, no thrill of either joy or pain.

"This has been such a pleasant time for me, Marion," he said in his caressing tones; "after all the fret and worry and excitement of my life, this rest, with you to take care of me, has been a very Eden. What it would have been without you I can't say, but you have made it paradise. You can understand that, can't you?—and you can understand how—how irresistible it was—how I could not help enjoying it to the full? To get back to the old relations with you once more was too tempting; your voice, the touch of your hand, seemed to make me young and strong, and hopeful once more. Perhaps it wasn't quite right, but if you only knew how sweet you are you would soon forgive me. And if I had come home earlier, before I had gone so far with Mrs. Durrant, it might have been different."

Jack thought it kind to "let her down gently," as he phrased it in his mind, and looked upon a compliment as a strong cord wherewith to do so.

"But—well—it's practically all arranged. I don't know that I have said much, but she has assumed that it is all as she wants. And the fact is, I have made rather a mess of things; nothing seemed to prosper with me, and now my health is gone, as you see. She is rich; and she gets a husband whom she admires, and who is white—old Durrant was an Eurasian, and she is touchy about her own mixed blood—and I get her money; and so, now that we have learned the world's lesson of taking just what we can get, it's the best thing for both of us. You see that, don't you—dear?"

He added the last word diffidently, expecting it to be followed by an outburst of indignation or a torrent of tears; but the truth was, that though he had hoped when Marion came in that she would not make a "scene" when he vouchsafed his explanation, he began to feel that a scene would be more endurable, more flattering, than this calm, patient hearing which was being granted him. Marion's stillness suggested that she was analysing what he said, and Jack Holtayne's statements were not adapted to analysis.

Even now she only answered,—"I see, at least, that you think it the best thing," without even a suspicion of sarcasm in her voice.

Jack was a little disappointed.

"It isn't as if you were a frivolous, selfish girl, who would take it to heart," he went on, explaining himself not very happily. "You have higher things to keep in your mind than a foolish fellow who loved you 'not wisely but too well'; yours is such a noble life, and you are so absorbed in it that—that—"

"We'll omit 'that,'" interpolated Marion.

"I shall always love you best," he assured her. "I daresay I shall think of you long after you have forgotten me. And if you marry Brandreth—"

"Let us omit that also," she said firmly.

"Oh! if you like. But say that you forgive me, that you don't grudge me my few days' happiness."

"There is nothing to forgive," she said, rising from her chair and facing him, but speaking in her usual gentle tone; "you have more than paid for any gratification I have offered you by effacing from my mind an illusion which had marred too long the happiness and usefulness of my life."

Holtayne looked less contented than might have been desired.

"Have you nothing kinder to say to me than that?" he asked fretfully, seeking to the last for some salve to his vanity.

"Mrs. Durrant seems to be of a simple and generous nature, and she is evidently attached to you; be kind to her."

Jack felt this to be an unsatisfactory farewell, but it was all he could elicit from her; and till he went away, two days afterwards, in charge of his betrothed, she resolutely confined her conversation to matters relating to his health. She was again Nurse Marion, and nothing more.

---

Chapter VIII.—Conclusion.

Marion was glad to get back to the hospital. The routine of her work there, which left her so few idle moments, was the best anodyne for the indignation that seemed to burn like poison in her every vein. She did not mourn for Jack Holtayne; for that fickleness which she now perceived to be indeed a too complete fidelity to his own selfishness; nor for the insult involved in making her a toy to beguile his idle hours. These things had inflicted a shock to her; but the very unworthiness of the man she had loved, brought a reaction that made the wound he had inflicted slight.

It was another cause that kept her face so pale and haggard, and made her spirit bitter to her old lover. The true sorrow lay in what she had lost for his sake. A good man had offered her his love, and she had not merely refused it, but had treated him with injustice and insult. She had thrown away the chance of true happiness for a will-o'-the-wisp, intangible and worthless; and though she admitted her fate to be just, it was none the less hard to bear.

Dr. Brandreth was a man of systematic ways. He let the thirty-one days of probation pass before he spoke again to Marion of anything but the work in which their common interest lay. She said to herself that if he had not forgotten that he had ever sought her for his wife, he wished her to forget that he had ever been guilty of such a folly, and she felt that she deserved no better lot than to be thus silently let alone.

But the day after the month had elapsed he sent for her, and asked quietly, as if alluding to a question of the minute before,—"Well, Marion, what is your final answer to me?"

"Do you still want an answer?" she demanded, bitterly. "You cannot care for me now: you must despise my folly, my infatuation too much."

The Doctor smiled as he looked at her downcast head, and though there was some humour in his smile, there was more tenderness. He answered her passion somewhat lightly, at least in appearance.

"I am a man of few impressions," he said, "but these few are permanent; and my love for you is the most firmly fixed of them. Nor do I see why I should despise you for fidelity, however misplaced. I am not sure that the woman who can most readily and accurately perceive a man's unworthiness is by any means the most lovable; I may make some demand on love's blindness myself some day. Dear," he added in a graver tone, "surely you do not mean to let the dream which has already darkened so many years of your life, sadden the remainder. Put it aside; be my wife, and let me try to make you happy. And even if you do not love me at first——"

But here Nurse Marion interrupted him, in a shy whisper, with the only coquettish speech she ever made.

"Are you quite sure I don't?" said she.

